# New Advances in Urogynecology

**DOI:** 10.1155/2012/453059

**Published:** 2011-12-18

**Authors:** Lior Lowenstein, Peter L. Rosenblatt, Hans Peter Dietz, Johannes Bitzer, Kimberly Kenton

**Affiliations:** ^1^Department of Obstetrics and Gyenceology, Rambam Health Care Campus, Haifa, Israel; ^2^Ruth and Baruch Rappaport Faculty of Medicine, Technion—Israel Institute of Technology, Haifa, Israel; ^3^Division of Urogynecology and Reconstructive Surgery, Mount Auburn Hospital, Harvard Medical School, Cambridge, MA, USA; ^4^Department of Pelvic Reconstructive Surgery and Urogynaecology, Sydney Medical School Nepean, Penrith, NSW, Australia; ^5^Department of Obstetrics and Gynaecology, University Hospital Basel, Basel, Switzerland; ^6^Division of Female Pelvic Medicine and Reconstructive Surgery, Departments of Obstetrics & Gynecology and Urology, Stritch School of Medicine, Loyola University Chicago, Maywood, IL, USA

Pelvic floor disorders, including urinary incontinence, pelvic organ prolapse (POP), and bowel dysfunction, affect millions of women worldwide resulting in considerable cost and quality of life impact. One-third of all women will suffer from these disorders at some point in their lives [1–5]. Significant research efforts are underway to improve our understanding of the pathophysiology, optimal evaluation, and effective treatment for women with pelvic floor disorders. More than ever before, research is providing meaningful developments into etiologies and novel treatment modalities. Additionally, researchers advance our understanding of improved methods for evaluating treatment outcomes, including patient-reported outcomes. These advances are largely due to the efforts of an increasing number of clinician-scientists who design and conduct high-quality clinical trials and translational studies. In addition to learning more about basic pathophysiology, recent technical advances offer excellent treatment efficacy with reduced morbidity. This work is facilitated by the efforts of multidisciplinary teams composed of a widening group of pelvic floor specialists, including radiologists, physiotherapists, urologists, and urogynecologists. The clinical advances in urogynecology are advancing rapidly and will improve the well-being of millions of women who suffer from pelvic floor disorders.

The main focus of this special issue is on new and existing diagnostic and treatment methods for pelvic floor disorders. The articles summarize current approaches to the treatment of these disorders and look into the future by discussing possible novel interventions for the treatment of pelvic floor dysfunction.

The first paper of this issue, published by a group of clinicians from The Netherlands, explores the association of POP severity and subjective pelvic floor symptoms. As one might expect, presence of POP on exam was associated with patient-reported symptoms of prolapse and voiding dysfunction, but not with urinary incontinence or defecatory symptoms. The second paper evaluates the role of pessary trial in predicting postoperative outcomes of occult stress urinary incontinence. The authors suggest that pessary trial is an effective method to evaluate POP patients for occult stress incontinence, as 20% of patients with occult stress incontinence were identified by pessary trial alone. The third paper presents a comparative study between two common methods to evaluate afferent neural function in the lower urinary tract. The current perception test is becoming increasingly important in diagnosing abnormalities of afferent neural pathways. Since these neurologic problems may contribute to certain pelvic floor disorders, it is important to establish the best methods for these neural changes. The authors conclude that the method of levels is superior to the method of limits when evaluating current perception thresholds in the lower urinary tract. The fourth manuscript reviews a new treatment for stress urinary incontinence, transurethral radio frequency treatment of the bladder neck and proximal urethra. Radio frequency is thought to reduce funneling of the bladder neck through the denaturation of submucosal collagen, with a resultant reduction in tissue compliance and increased Valsalva leak point pressure. The authors conclude that radio frequency is an effective conservative treatment for stress incontinence with few side effects.

In the fifth paper, from the Cleveland Clinic, an animal model was used to evaluate whether intravenously injected mesenchymal stem cells home to pelvic organs after simulated childbirth injury. The findings of this interesting paper provide evidence that intravenous administration of mesenchymal stem cells may be used as an early intervention to repair injuries to the levator ani muscles and both urethral and ani sphincters, thus preventing future pelvic floor disorders.

The sixth paper presents results of an Israeli survey evaluating trends among Israeli urogynecologists regarding the routine use of mesh. The use of mesh in vaginal prolapse surgery is a hot topic, especially since the last safety notification published by the FDA on July 13, 2011 regarding possible adverse events following the use of vaginal mesh. Ironically, the use of mesh among Israeli urogynecologists increased significantly over the last two years. Though the data regarding the efficacy and safety of vaginal mesh is still lacking, the popularity of this method continues to rise. One possible explanation for this discrepancy is that Israeli physicians practice medicine in an environment characterized by innovation and scientific progress. Until studies with higher levels of evidence prove the efficacy of these treatments, more caution should be advised in the application of this yet unproven technology. The next paper published by Dr. K. T. Downing is a comprehensive review article regarding the progress of treatment of uterine prolapse from ancient times up to the present day. This article is especially relevant for those who are looking for new advances in medicine. As was stated previously by George Santayana, “Those who cannot remember the past are condemned to repeat it.”

Last but not least is a paper published by a group of researchers from Spain who evaluated the level of training of residents in obstetrics and gynecology in the management of perineal tears that occur during assisted vaginal delivery. Almost all of the respondents indicated that more training in this specific area is necessary (98%). As Ralph Waldo Emerson once stated, “Skill to do comes of doing.”

Finally, in this special issue, the reader will conveniently find a comprehensive summary of the state-of-the-art diagnostic strategies and new advances in urogynecology. 

**Lior Lowenstein** **Lior Lowenstein** **Peter L. Rosenblatt** **Peter L. Rosenblatt** **Hans Peter Dietz** **Hans Peter Dietz** **Johannes Bitzer** **Johannes Bitzer** **Kimberly Kenton** **Kimberly Kenton**
